# Microscopy of Minute Organisms

**Published:** 1872-07

**Authors:** S. P. Cutler


### SELECTED ARTICLES.

### *Microscopy of Minute Organisms.*

BY S. P. CUTLER, M.D., D.D.S.

Read before the New Orleans Academy of Science.

Take some of the decayed matter from a decayed tooth—the greenish slimy scum on the surface of filthy and neglected teeth; or matter lodged between the teeth, and the accumulation of tartar on them, and put it into the animalcule cage with a drop of water, and place under the microscope and look down.

At first the inexperienced observer will discover only confused masses of matter scattered over the field; but, on close observation, definite, minute, dark points, will be seen in constant vibratory motion, scarcely leaving their places, but a motion that never ceases. This motion is brought about by some invisible means not recognizable. These minute forms are apparently globular, or nearly so, varying in size by actual measurements by myself, from the 1–60,000th of an inch to the 1–10,000th of an inch in diameter, these measurements being the recognizable limits. I have seen none larger, nor could I make out any smaller distinctly. By improved methods of illuminating the objects, they may be recognized still smaller. Five hundred diameters, or thereabouts, shows them to best advantage.

In some specimens, threads of *Odium Albican* may be seen, varying in length, like minute white threads, about 1–12,000th of an inch in diameter, and indefinite lengths. Besides these, two other forms may be made out. One would appear to be composed of three of these globular points, united lineally, called *vibrois* by Darwin, *gemmules* by others, *denticolæ* by some; occasionally *leptothrix*, or serpent forms, may be seen, the head measuring 1–10,000th of an inch in diameter, tapering to a point, many times longer than their diameter, swimming with serpent like motions and rapidity. These are not numerous—in many specimens wanting.

There is another quite constant form, slipper shape, or like a blunt wedge, much larger than the others, with sluggish but constant motion, larger, and measuring about 1–6,000 of an inch, their length being about double their thickness. I cannot say whether they are any kin to the others or not, probably not; they no doubt belong to an aristocratic family, or one of the first families in the microcosmic dominion.

The *oidium* may be seen contracting so as to produce a jerking kind of motion when a number of them are attached to any fragments of matter, by contracting suddenly in slow repetition, but continuous at times; at other times at rest. These may be parasitic plants, or may be animals.

Wax from the ears, and dried mucus from the nose, contain them in great numbers, besides epitheliel cells and other matters dried.

Sputa from the lungs, in case of cough, contain them in great quantities, more especially in consumption, and all chronic coughs.

A specimen of pus, running from the outside through an opening from a fractured jaw, contained but few animalcules of any kind; a case of four months' standing. A piece of alveolus removed about the same time, half an inch in length, contained myriads of the smallest globular forms.

The specimen of pus was allowed to remain on the glass in the moist chamber twenty-four hours, which developed moderately plentiful. I refer mainly to the globular form, which are always present whenever any of the others are seen, and in many cases where the others are not seen, and are always much the most numerous in all cases.

Numerous specimens of decayed teeth, that had been out of the mouth for several years, were examined, and all found to contain them in great numbers. So soon as water was put into the cavity and stirred around with some pointed instrument, and some of the milky-looking matter put in the cage and examined, they became as active at once as those from a fresh specimen just from the mouth, in every instance.

A number of pulps were examined from teeth that had ached, and were more or less inflamed, and in various stages of decomposition, all of which contained them in great abundance; in some instances more numerous than others. Some were intensely injected with blood. These contained the most, as a rule.

The pulp chambers from a number where there was a healthy pulp at the time the teeth were removed, all contained them in greater or less quantities.

Several more perfectly healthy and not inflamed fresh pulps were examined, and all found to contain them.

A decayed tooth that had been out of the mouth over twenty-five years, contained them in as great quantities as any other specimen.

A lot of sound teeth that had been kept in alcohol—for the purpose of replacing lost ones—about fifty years, from reliable data, on splitting open several of them, and examining the alcohol from the nerve chambers, all contained them in as lively a condition as any other specimens, there being no signs of pulp in them. The alcohol also contained them. These teeth came from France, so supposed, and were most likely taken from dead subjects, from the dissecting rooms. There were some fine globules, as large as the 1–8,000th of an inch. These had a spot in the centre, resembling a nucleus. None of the others were any larger than any of the other specimens; no difference in any respect discoverable. They lived in the alcohol all this time; no doubt the alcohol had been renewed oftentimes; *only the one form seen.*

A specimen of pus, from a running fistulous opening in the cheek, where a large portion of bone had been removed several years since, from necrosis, contained them in great numbers.

I split open a perfectly healthy and sound incisor tooth that had been out about fifteen years, the dried pulp perfectly white, and on adding a drop of water, and then removing the pulp, it was found to contain myriads of them.

I examined dozens of other decayed teeth, with uniform results—especially small globular forms were present.

Specimens of human and other blood, catamenial flux, numerous specimens of earth, carmine, musquito, prepared chalk and other cosmetics, chalk, plaster-of-Paris, shells pulverized, sulphate of manganese, arsenicum, arsenous acid, per oxide of lead, all more or less full.

Ashes from a burning grate, ashes from a burning cigar, blue litmus, cork dissolved in caustic potash, pulverized egg shell, bones, pitch, and linen from Egyptian mummy, pulverized marble, brick-dust, all contain them, varying somewhat.

Starch, powdered and loaf sugar, do not contain any or I failed to find them.

Orange pulp, pine and seeds, full of the globular forms.

Beef broth (fat of,) and muscles, and mutton, and all other meats; yolks and whites of eggs—all are full of them.

Fresh biscuit, rice, onions, sweet and Irish potatoes, old ham, beans, lettuce stalk, cabbage (rib of leaf), celery stalk, all contain them.

A specimen containing myriads of them I put into a vulcanizing oven, with water, and raised the heat up to 320°, and kept it at that for one hour, which produced no effect on them.

I tried to kill them with sulphuric acid, caustic potash, concentrated then diluted; also, oxalic acid, chloroform, concentrated ammonia, creasote, chromic acid, lunar caustic, chloride of zinc; all failed to affect them. I have never been able to destroy them in any way.

I examined numerous scabs and other diseased matters of various kinds. All contain them in great quantities.

(*To be continued.*)

